# Global trends in pediatric burn injuries and care capacity from the World Health Organization Global Burn Registry

**DOI:** 10.3389/fped.2022.954995

**Published:** 2022-07-19

**Authors:** Kelly C. Jordan, Jane L. Di Gennaro, Amélie von Saint André-von Arnim, Barclay T. Stewart

**Affiliations:** ^1^Division of Pediatric Critical Care Medicine, Department of Pediatrics, Seattle Children's Hospital, University of Washington, Seattle, WA, United States; ^2^Department of Global Health, University of Washington, Seattle, WA, United States; ^3^Division of Trauma, Burn, and Critical Care Surgery, Department of Surgery, University of Washington, Seattle, WA, United States

**Keywords:** global health, pediatric burn injuries, pediatric critical care, global burn registry, low-middle income countries (LMIC), low resource settings, burn

## Abstract

**Background:**

Burn injuries are a major cause of death and disability globally. The World Health Organization (WHO) launched the Global Burn Registry (GBR) to improve understanding of burn injuries worldwide, identify prevention targets, and benchmark acute care. We aimed to describe the epidemiology, risk factors, and outcomes of children with burns to demonstrate the GBR's utility and inform needs for pediatric burn prevention and treatment.

**Methods:**

We performed descriptive analyses of children age ≤ 18 years in the WHO GBR. We also described facility-level capacity. Data were extracted in September of 2021.

**Results:**

There were 8,640 pediatric and adult entries from 20 countries. Of these, 3,649 (42%) were children (0–18 years old) from predominantly middle-income countries. The mean age was 5.3 years and 60% were boys. Children aged 1–5 years comprised 62% (*n* = 2,279) of the cohort and mainly presented with scald burns (80%), followed by flame burns (14%). Children >5 years (*n* = 1,219) more frequently sustained flame burns (52%) followed by scald burns (29%). More than half of pediatric patients (52%) sustained a major burn (≥15% total body surface area) and 48% received surgery for wound closure during the index hospitalization. Older children had more severe injuries and required more surgery. Despite the frequency of severe injuries, critical care capacity was reported as “limited” for 23% of pediatric patients.

**Conclusions:**

Children represent a large proportion of people with burn injuries globally and often sustain major injuries that require critical and surgical intervention. However, critical care capacity is limited at contributing centers and should be a priority for healthcare system development to avert preventable death and disability. This analysis demonstrates that the GBR has the potential to highlight key epidemiological characteristics and hospital capacity for pediatric burn patients. To improve global burn care, addressing barriers to GBR participation in low- and low-middle-income countries would allow for greater representation from a diversity of countries, regions, and burn care facilities.

## Introduction

Burn injuries are a leading cause of death and disability among children globally (1–4). The World Health Organization (WHO) estimates that burn injuries account for ~180,000 deaths annually and are the fifth most common cause of non-fatal childhood injuries (5). The burden of child mortality due to burn injuries reflects the inequity of risk factors and care capacity, as rates of child deaths from burns are over seven times higher in low- and middle-income countries when compared to high-income countries (5). Due to the high risk of mortality for burn-injured patients, the physical and psychosocial impacts on people living with burn injury, and the critical lack of both burn and pediatric care capacity globally, the causes and treatment of pediatric burns deserve special focus (6–8).

Acute pediatric burn management ranges from simple outpatient treatment to complex emergency, critical care, and surgical interventions (9). For children with more severe injuries, critical and surgical care capacity consisting of pediatric airway management, sedation for frequent wound care, surgical debridement and reconstruction, tailored nutritional support, and multidisciplinary rehabilitation services are vital for survival and successful long-term outcomes. However, comprehensive resources for pediatric burn care are not readily available globally (10). A study of Ugandan anesthetists demonstrated that only 13% of those surveyed had the supplies necessary to deliver safe anesthesia to a child (11). Reports from Ghana, Nepal, Mexico, India, and Vietnam suggest that lack of emergency and critical care capabilities, and specialty trained personnel, are common barriers to successful outcomes (6, 12, 13). Outside of high-income countries, availability of burn care resources are variable and often limited (4, 14, 15). Although a significant body of research focuses on burn prevention, there is a dearth of information about the burn care capacity in a diversity of locations, specifically the unique needs of burn-injured children.

Burn injuries can have significant psychological, social, and economic impacts on a child's life and the lives of their caregivers (16). Data regarding pediatric global burn trends is inconsistent and frequently extrapolated from single-center or country-specific studies that fail to provide a clear understanding of the global problem (15, 17). To address gaps in epidemiological understanding of global burn trends and availability of key resources, WHO established the Global Burn Registry (GBR), a central platform for the collection and analysis of burn injuries worldwide. The GBR was designed to define global burn injury epidemiology, support prevention initiatives, and benchmark quality improvement activities (18). Our aim was to describe the epidemiology of and capacity for treating pediatric burn injuries worldwide as represented by the WHO GBR.

## Materials and methods

### Data source—Global Burn Registry

The WHO launched the Global Burn Registry in 2017 to improve understanding of burn injuries and care capacity worldwide. The validated data collection form utilized by participating health facilities was developed through the collaboration of experts from the WHO, International Society for Burn Injuries (ISBI), and Global Alliance for Clean Cookstoves. Participating facilities utilize a data collection toolkit, initially recorded on a paper form, and later electronically transcribed and uploaded into the GBR. The finalized version of the data collection form, implementation specifics, and ideal uses was published by the group (18). The GBR is continuously updated and stored on network servers located in Geneva, Switzerland at WHO headquarters. Public and private versions of the registry data are available. Public data are modified to remove potential patient identifiers. For this study the public WHO GBR data were accessed *via* https://www.who.int/teams/social-determinants-of-health/safety-and-mobility/burns/global-burn-registry on September 17, 2021.

### Data analysis

Data were extracted for patients aged ≤ 18 years and were grouped into age categories based on childhood developmental stages: infants (<1 year), toddlers and preschoolers (1–5 years), grade schoolers (6–12 years), and adolescents (13–18 years) (19). Descriptive statistics were used to highlight patient demographics, burn injury risk factors, burn injury characteristics, outcomes (including in-hospital death, discharge with disability), and facility capabilities (access to critical care, an operating theater, nutrition services, physical therapy and rehabilitation services, blood transfusion, and internet connectivity).

## Results

### Patient demographics

The GBR contained data from 8,640 patients with burn injuries from 20 countries: Argentina, Chile, China, Estonia, Ethiopia, India, Iran, Kenya, Laos, Mexico, Nepal, Nigeria, Pakistan, Peru, Philippines, Russian Federation, Saudi Arabia, South Africa, Sri Lanka, and Tanzania. Of entries in the GBR, 3,649 (42%) were children. The mean age of this group was 5.3 years (SD 5.01). Children aged 1–5 years comprised 62% (*n* = 2,279) of the pediatric cohort and 26% of all burn injuries recorded in the GBR. School aged children (age 6–12 years) were the next most frequently burn-injured group with 20% (*n* = 732) of pediatric burns recorded by the GBR, followed by adolescents (age 13–18 years) at 13% (*n* = 487). The overwhelming majority of pediatric patients were from centers in middle-income countries (99%, *n* = 3,628). More boys (60%) were reported than girls (40%) (Table 1).

**Table 1:** Pediatric patient demographics.

	<**1 year**		**1–5 years**		**6–12 years**		**13–18 years**		≤ **18 years**	
	***n***	**%**	***n***	**%**	***n***	**%**	***n***	**%**	***n***	**%**
Number of patients	151	4	2,279	62	732	20	487	13	3,649	42
**Sex**										
Female	57	38	939	41	292	40	176	36	1,464	40
Male	94	62	1,340	59	440	60	311	64	2,185	60
**National income level**										
High income	1	1	7	0	2	0	7	1	17	0
Middle income	150	99	2,271	99	728	99	479	98	3,628	99
Low income	0	0	1	0	2	0	1	0	4	0

### Pediatric burn injury etiology, characteristics, and outcomes

We defined three burn categories based on data in the GBR which included flame, scald, and other (i.e., burns that were chemical, cooling, electrical, friction, or hot surface related). For all pediatric patients, scald burns were the most common (involving contact with hot liquid, steam, or gas) and accounted for 62% of all pediatric burns reported (*n* = 2,261) followed by flame burns (27%, *n* = 997). Children 1-5 years sustained the highest proportion of scald burns (80%, *n* = 1,826); flame burns only accounted for 14% of burns in this age group (*n* = 320). Children older than 5 years (grade schoolers and adolescents, *n* = 1,219) more frequently sustained flame burns (52%, *n* = 628) followed by scald burns (29%, *n* = 348). Adolescents (age 13–18 years, *n* = 487) had the highest frequency of admissions for flame burns at 60% (*n* = 292) as well as burns of any age group being categorized as “other” (25%, *n* = 122) (Table 2).

**Table 2:** Pediatric burn injury etiology, characteristics, and outcomes.

	<**1 year**		**1–5 years**		**6–12 years**		**13–18 years**		≤ **18 years**	
	***n***	**%**	***n***	**%**	***n***	**%**	***n***	**%**	***n***	**%**
Number of patients	151	4	2,279	62	732	20	487	13	3,649	100
**Type of burn**										
Flame	49	32	320	14	336	46	292	60	997	27
Scald	87	58	1,826	80	275	38	73	15	2,261	62
Other	15	10	133	6	121	17	122	25	391	11
**Severity of burn injury**										
Minor burn <15% TBSA^a^	69	46	1,124	49	370	51	178	37	1,741	48
Major burn ≥15% TBSA	82	54	1,155	51	362	49	309	63	1,908	52
Associated inhalation injury	18	12	93	4	64	9	87	18	262	7
**Burn location**										
Head and neck	74	49	920	40	287	39	268	55	1,549	42
Trunk	90	60	1,547	68	437	60	320	66	2,394	66
Arms	86	57	1,266	56	377	52	314	64	2,043	56
Hands and wrist	68	45	755	33	288	39	284	58	1,395	38
Legs	86	57	1,196	52	449	61	324	67	2,055	56
**Disposition**										
Death	17	11	160	7	67	9	111	23	355	10
Home with disability	14	9	123	5	62	8	45	9	244	7
Home without disability	104	69	1,862	82	552	75	291	60	2,809	77
Other (transferred, left AMA^b^, unknown)	16	11	134	6	51	7	40	8	241	7
**Care utilization**										
Presentation to care <2 h	105	70	1,492	65	444	61	298	61	2,339	64
2– <8 h	30	20	575	25	198	27	133	27	936	26
8–24 h	9	6	164	7	52	7	35	7	260	7
>24 h	7	5	48	2	38	5	21	4	114	3
Surgery at index hospital	58	38	989	43	422	58	281	58	1,750	48
Length of stay, days (median, IQR^c^)	9 (5.1–17.8)		10 (5.3–17.9)		10.8 (5.4–17.8)		10.2 (4.9–20.5)		10.2 (5.2–18.1)	

^a^*Total body surface area*.

*^b^Against medical advice*.

*^c^Interquartile range*.

The severity of burns also noticeably varied across age groups. We defined major burns as affecting a total body surface area (TBSA) ≥15% (20). More than half of children (52%, *n* = 1,908) sustained a major burn. Adolescents (age 13–18 years, *n* = 487) sustained more severe burns more frequently than other age groups (63% with major burns, *n* = 309) and commonly had associated inhalation injuries (18%, *n* = 87) (Table 2).

In terms of burn location, the most frequent area of the body injured was the trunk (66%, *n* = 2,394); 56% of children sustained burns on their arms or legs (*n* = 2,043 and *n* = 2,055). Hand and wrist burns were most frequent amongst adolescents (58%, *n* = 284) and affected 38% (*n* = 1,395) of all pediatric patients. Almost half of patients (48%, *n* = 1,750) received surgery for wound closure while hospitalized. The median length of stay was 10.2 days (IQR 5.2–18.1 days). Adolescents experienced the highest frequency of death (23%, *n* = 111). For all pediatric patients in the GBR, 10% died during hospitalization (*n* = 355); 7% of children (*n* = 244) were discharged home with a disability (as defined by the contributing burn care facility) (Table 2).

### Burn facility characteristics

The majority of children (64%, *n* = 2,339) were cared for by the contributing healthcare facility within 2 h of their injury, and 90% (*n* = 3,275) arrived or were transferred to a contributing hospital within 8 h of injury. Most children were treated in government-operated hospitals (86%, *n* = 3,143). Participating facilities reported their capacity in terms of critical care (e.g., distinct unit or beds with more care than those on general wards), access to an operating theater, nutrition services, physical therapy and rehabilitation services, blood transfusion capability, and internet connectivity. Of these centers, 23% (*n* = 832) had limited availability of critical care services. Reliable access to an operating theater was reported by 92% (*n* = 3,375) of participating facilities. There was limited blood transfusion capability in 19% (*n* = 703) and physical therapy and rehabilitation services in 14% (*n* = 502) of facilities. Almost a third of facilities (30%, *n* = 1,113) reported general nutrition capability with enteral feeds and nasogastric feeds, and 70% (*n* = 2,536) indicated advanced nutrition capability (e.g., person-centered nutritional support program) (Table 3).

**Table 3:** Burn facility characteristics.

	<**1 year**		**1–5 years**		**6–12 years**		**13–18 years**		≤ **18 years**	
	***n***	%	***n***	%	***n***	%	***n***	%	***n***	%
Number of patients	151	4	2,279	62	732	20	487	13	3,649	100
**Ownership**										
Government	135	89	1,963	86	627	86	418	86	3,143	86
Private	16	11	316	14	105	14	69	14	506	14
**Critical care**										
Reliable access	83	55	1,681	74	606	83	446	92	2,816	77
Limited access	68	45	598	26	126	17	41	8	833	23
**Operating theater**										
Reliable access	124	82	2,124	93	675	92	452	93	3,375	92
Limited access	27	18	155	7	57	8	35	7	274	8
**Nutrition services**										
Advanced	90	60	1,529	67	530	72	387	79	2,536	70
Enteral/nasogastric feeds	61	40	750	33	202	28	100	21	1113	30
**Physical therapy/rehabilitation services**										
Reliable access	97	64	1,978	87	628	86	444	91	3,147	86
Limited access	54	36	301	13	104	14	43	9	502	14
**Blood transfusion capability**										
Full	96	64	1,860	82	579	79	411	84	2,946	81
Limited	55	36	419	18	153	21	76	16	703	19
**Internet connectivity**										
Reliable	111	74	1,727	76	591	81	410	84	2,839	78
Intermittent	40	26	552	24	141	19	77	16	810	22
**Annual burn admissions**										
<50–100	63	42	791	35	283	39	184	38	1,321	36
>100–300	60	39	1,102	49	273	37	144	29	1,578	43
>300	28	19	386	16	177	24	159	33	750	21

## Discussion

The WHO GBR is an open-access database that was created to improve understanding of global burn injury epidemiology, identify key prevention targets, and inform quality improvement measures through a standardized and centralized data collection system. This study utilized the WHO GBR to describe the epidemiology of global pediatric burns and evaluate it as a tool for monitoring facility capacity when it comes to the care of burn-injured children. The GBR demonstrates that children (age ≤ 18 years) represent almost half of burn injuries globally (42%) and young children (age 1–5 years) comprise the greatest proportion of pediatric burn injuries (62% of children ≤ 18 years, 26% of all GBR entries). Approximately half of the children in the GBR sustained a major burn (≥15% total body surface area, 52%) and required surgical wound closure (48%). The volume and severity of pediatric burn injuries warrants robust pediatric emergency, critical care, and surgical capacity, however current GBR data demonstrates that critical care capacity is limited in almost a quarter of participating facilities (23%) the majority of which are in middle income countries. Given the prevalence of childhood burn injuries as demonstrated by current GBR data, dedicated efforts to improving participation in the GBR from a diversity of regions and healthcare facilities, as well as evaluating pediatric emergency, critical, and surgical care systems are essential for improving pediatric burn care globally.

The GBR is the first database to allow global monitoring of pediatric burn injury trends, outcomes, and burn care capacity *via* participating facilities. The GBR demonstrates that the pediatric burn trends observed are consistent with prior smaller, single-center studies that reported young children aged <5 years are the most frequently burned (21), and scald injuries are the most common type of pediatric burn followed by flame burns, particularly in adolescents (1, 22, 23). Current GBR data shows that over half of pediatric patients sustain major burns that would necessitate treatment in a burn center with pediatric emergency and critical care services; however, almost a quarter of pediatric patients were treated in facilities with limited availability of adult critical care, let alone pediatric-specific capabilities. In resource limited settings, access to pediatric critical care providers is limited (10, 24) and the concept of dedicated burn care is often not recognized, with most burn injured patients receiving care from hospital specialists or general surgeons with no specific burn injury training (14). Delays in resuscitation also lead to increased mortality; a study of pediatric burn care in Sub-Saharan Africa found that in Nigeria administration of intravenous saline and dextrose were based on availability and were often not given to children with major burn injuries (25). Pediatric-focused assessments of emergency, surgical, and critical care capacity can be helpful in documenting current equipment and personnel needs at the facility level (6). The availability of personnel trained to employ pediatric emergency care equipment is an important component of effective care and resource utilization (12, 24). Evaluation of pediatric burn care capabilities at the facility level could help improve not only pediatric burn outcomes, but pediatric trauma, respiratory failure, diarrhea, and sepsis outcomes as well; given that shared capabilities are required to manage these emergency conditions. Given the prevalence of child burn injuries globally, and the frequent absence of trained personnel and pediatric specific equipment (10, 24), facility assessment of pediatric capacity is another facet of equitable burn care that the GBR could explore.

### Challenges facing the GBR

Although there is suggestion that burn care and outcomes for children are improving globally (26), the geographic and socioeconomic distribution of high-quality burn care remains inequitable (4, 14, 27, 28). Currently, children under five years of age in sub-Saharan Africa have over twice the incidence of burn deaths compared to children under five worldwide (5). The GBR has the potential to gather and monitor epidemiologic burn data from a diverse range of countries and facilities in response to concerted efforts to improve global trauma care (e.g., dissemination of WHO Emergency Care System products, global promulgation of Advanced Trauma Life Support, uptake of Interburns Quality Improvement Framework at national levels). Currently, the GBR contains data from only 20 countries, with an even smaller number reporting the bulk of current burn data. Additionally, most of the facilities participating in the GBR are in middle-income countries. Thus, facilities in low- and low-middle-income countries, the most likely to contribute to preventable morbidity and mortality, are not being captured by the GBR. It is unclear what the most pressing barriers to participation are currently. There are potential language barriers, as the form is only available in English, French, and Spanish. There are also potential barriers related to human capacity, as healthcare providers in resource limited settings are often stretched into multiple roles in an already overburdened system (25), or due to limited access to reliable internet, as both are necessary to gather and upload data into the GBR. Shifting data entry responsibilities to staff outside of nurses and doctors and improving access to the GBR database *via* mobile devices might improve participation in certain settings. For facilities with already strained resources, efforts to understand demand-side incentives might also contribute to more rapid and broader uptake of GBR involvement (e.g., including GBR contribution as verification criteria, linking contributions to capacity building initiatives). Assessment of the resources to improve the adoption, penetration, and sustainability of GBR in low- and low-middle-income countries should be pursued.

### Improving pediatric burn care

In addition to the challenges of participating in the GBR, there is also a lack of pediatric-focused assessments that could inform how to improve facility capacity for pediatric burn care. At its inception, the goal of the GBR was to move from a fragmented approach to a standardized, global data collection system to improve public health measures. However, high-quality pediatric burn care requires specialized equipment, monitoring, and personnel that the GBR is not currently capturing (e.g., availability of multiple sizes of supplies, pediatric-specific care knowledge and skills). According to the GBR, over half of the pediatric cohort sustained a burn that would warrant transfer to a burn center with a pediatric intensive care unit, specialized surgical care, and rehabilitation (at a minimum, a volume requirement of >100 children per year) (20, 29). Over a third of facilities in the GBR treating pediatric patients (36%) would not qualify as a burn center according to these criteria. The GBR describes availability of critical care at participating facilities and current data demonstrates insufficient adult critical care capacity. Including availability of pediatric critical care would better inform pediatric burn care capabilities for children with the highest risk of mortality. This would not only establish resuscitation and airway management expertise but would also encompass advanced skill related to pediatric specific post-operative care, procedural sedation and analgesia, nutrition needs in the setting of critical illness, and comfort with patients spanning a broad range of developmental stages.

Part of acute burn care involves surgical debridement and reconstruction (e.g., skin grafting, soft-tissue flap coverage). Children are at increased risk of morbidity due to contractures after a burn as they are smaller and have thinner skin, and a similar injury will cause a deeper burn and more extensive skin loss compared to an adult (22). However, many centers in low-income countries are lacking critical resources including operating time, dedicated burn surgeons, anesthetists, and nurses (14, 25). Currently, GBR participants document access to an operating theater, indicating the potential presence but not the availability of surgeons and anesthetists, and currently there is no way of assessing their capability in terms of pediatric specific care. The capacity for pediatric general anesthesia is often limited in low-resource settings, this is commonly due to the lack of pediatric specific equipment and supplies in addition to human resources (11). A study of pediatric trauma supplies in Ghana showed a consistent scarcity of pediatric-sized items and lack of training when compared to adult-sized items (6). Potential indicators of basic pediatric anesthesia capabilities include an adequate oxygen supply, suction, pulse oximeter, but more specifically a range of pediatric face masks, laryngoscopes, endotracheal tubes, oropharyngeal airways, and breathing circuit (11).

Although equipment and human capacity can have significant impacts on acute burn care, preexisting malnutrition or anemia can also delay the decision to operate as nutritional status is an important consideration for successful burn recovery after surgery (25). Currently the GBR reports whether there are advanced nutritional services vs. general enteral feeding or nasogastric feeding capabilities. However, nutritional needs for children vary by age, with young children being at the highest risk of inadequate nutrition. This coupled with preexisting malnutrition can contribute to increased risk of morbidity and mortality from a burn injury (25). Early feeding in patients with severe burn injuries minimizes the effects of hypermetabolism and catabolism, reduces risk of malnutrition, promotes healing, and decreases length of stay in the ICU (30, 31). However, successful enteral and parenteral feeding is resource intensive, and assessing whether a pediatric patient received supplemental nutrition aids in capturing resource use and can help support burn service planning (32). Assessing a child's nutritional status with a height and weight measurement upon admission and discharge could inform a child's nutritional status at the time of a burn injury, this in addition to documentation of early enteral nutrition could highlight capacity needs for pediatric focused nutrition.

### Limitations and next steps

There are multiple limitations to discuss related to this work. First, participation in the GBR is voluntary and therefore not representative of all facilities or communities within a participating country (33). Similarly, a small number of middle-income countries are contributing the largest proportion of data to the GBR, which is currently lacking significant participation from both high-income and low-income countries. This limits the GBR from providing a comprehensive picture of global burn trends to the experience of a much smaller number of centers. Lastly, and most relevant to this study, pediatric specific care capacity information is not currently being collected by the GBR. In settings with limited resources and limited access to providers with specialized skills and equipment, management of pediatric burns constitutes a major challenge that warrants attention from the GBR. Table 4 proposes a pediatric module for assessing the unique capacity needs of burn injured children. Most of these data are already collected currently by the GBR and bolded text are examples of additional information that could inform pediatric burn care capacity in diverse hospital settings. Further exploration of this module would require additional discussion and piloting.

**Table 4:** Potential global burn registry pediatrics module.

**GBR domain**	**Minimum additional data elements**	**Aspirational information**
Epidemiology	Child ≤ 5 years old	
	Supervision (adult/child/none)	
	**Burn occurred within cooking area of home (yes/no)**	
Care utilization	Surgery at index hospitalization (yes/no)	**If no surgery performed, was it due to lack of: Surgeon (yes/no)**
		**Anesthesia (yes/no)**
		**Pediatric critical care (yes/no)**
Facility capacity	**Annual pediatric burn admissions (≤18 years old)**	
Critical care	**Pediatric critical care trained physicians(reliable/unreliable/none)**	**Patient required intubation within 24 h of admission (yes/no)**
	**Pediatric critical care trained nurses (reliable/unreliable/none)**	**Patient required unanticipated intubation/re-intubation (yes/no)**
		**Patient received vasopressor support (yes/no)**
		**Patient received antibiotics and vasopressor support due to sepsis (yes/no)**
Surgical Care	**Surgeon (reliable/unreliable/none)**	**If reliable surgeon presence: Burn surgeon (yes/no)**
	**Anesthesia (reliable/unreliable/none)**	**Discharge with contracture (yes/no)**
		**If reliable anesthesia presence: Pediatric anesthesia (yes/no)**
Nutrition	**Advanced nutrition services** **Enteral/nasogastric feeds** **Admission and discharge weight**	**If TBSA ≥15% was enteral/parenteral nutrition begun within 24 h of admission? (yes/no/NA)**
		**Did patient receive enteral or parenteral feeds?**
		**Middle Upper Arm Circumference (MUAC) % change admission to discharge**
Outcomes	**Death**	**Patient able to return to school (yes/no/unknown)**
	**Home with disability**	
	**Home without disability**	

## Conclusion

Children experience a large proportion of burn injuries globally and management of pediatric burns is demanding even in the most advanced settings. Current GBR data shows that pediatric patients make up almost half of burn injuries worldwide (42%) and young children (age 1–5 years) comprise the greatest proportion of pediatric burn injuries (62% of children ≤ 18 years, 26% of all GBR entries). Children with burn injuries commonly require unique emergency, surgical, and critical care capabilities, however this study reveals that there is still work to be done to improve capacity for quality comprehensive burn care globally. With greater representation from a diversity of regions and burn care facilities, better understanding of barriers to promulgation and implementation, and addition of modules for key populations for quality improvement initiatives, the GBR will be an excellent resource for monitoring global burn injury trends.

## Data availability statement

The original contributions presented in the study are included in the article/supplementary materials, further inquiries can be directed to the corresponding author/s.

## Author contributions

KJ conceived and designed analysis, performed analysis, and wrote the paper. JD conceived and designed analysis, contributed data and analysis tools, assisted with performing analysis, and assisted with writing the paper. AS assisted with analysis design and writing the paper. BS conceived and designed analysis, contributed data and analysis tools, and assisted with writing the paper. All authors contributed to the article and approved the submitted version.

## Conflict of interest

The authors declare that the research was conducted in the absence of any commercial or financial relationships that could be construed as a potential conflict of interest.

## Publisher's note

All claims expressed in this article are solely those of the authors and do not necessarily represent those of their affiliated organizations, or those of the publisher, the editors and the reviewers. Any product that may be evaluated in this article, or claim that may be made by its manufacturer, is not guaranteed or endorsed by the publisher.
